# Therapeutic validation and targeting of signalling networks that are dysregulated in intellectual disability

**DOI:** 10.1111/febs.16411

**Published:** 2022-02-28

**Authors:** Francisco Bustos, Greg M. Findlay

**Affiliations:** ^1^ Pediatrics and Rare Diseases Group Sanford Research Sioux Falls SD USA; ^2^ Department of Pediatrics Sanford School of Medicine University of South Dakota Sioux Falls SD USA; ^3^ The MRC Protein Phosphorylation & Ubiquitylation Unit School of Life Sciences The University of Dundee Dundee UK

**Keywords:** intellectual disability, neurodevelopmental disorders, post‐translational modification, signal transduction, stem cells

## Abstract

Intellectual disability (ID) represents a major burden on healthcare systems in the developed world. However, there is a disconnect between our knowledge of genes that are mutated in ID and our understanding of the underpinning molecular mechanisms that cause these disorders. We argue that elucidating the signalling and transcriptional networks that are dysregulated in patients will afford new therapeutic opportunities.

## Graphical abstract

Intellectual disability (ID) patients are diagnosed in the clinic and genetic testing identifies ID genes. Combining stem cell models with state‐of‐the‐art techniques to study the regulation and function of ID gene products allows dissection of novel signalling pathways that are disrupted by ID gene variants. This identifies molecular targets for therapies focused on restoring cell signalling to treat ID patients and improve cognitive function.

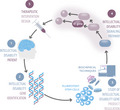

**Abbreviations**
DUB: deubiquitinase
ID: intellectual disability
PKU: phenylketonuria
PROTAC: proteolysis targeting chimera
SRPK: SRSF protein kinase
TOKAS: Tonne–Kalscheuer syndrome
XLID: X‐linked intellectual disability

## Introduction

Intellectual disability (ID) is a series of neurodevelopmental disorders with overlapping clinical features, including impaired adaptive and cognitive function. ID represents a major burden for patients, families and healthcare systems [1]. These conditions are a critical unsolved biomedical problem that affects an estimated 1–3% of the world population, especially children [2, 3, 4]. Therefore, there is an urgent need to develop novel therapies for ID, as most have no known cure [5]. We attribute the dearth of therapeutic strategies for ID to lack of knowledge as to the underlying molecular basis of these disorders.

**Fig. 1:**
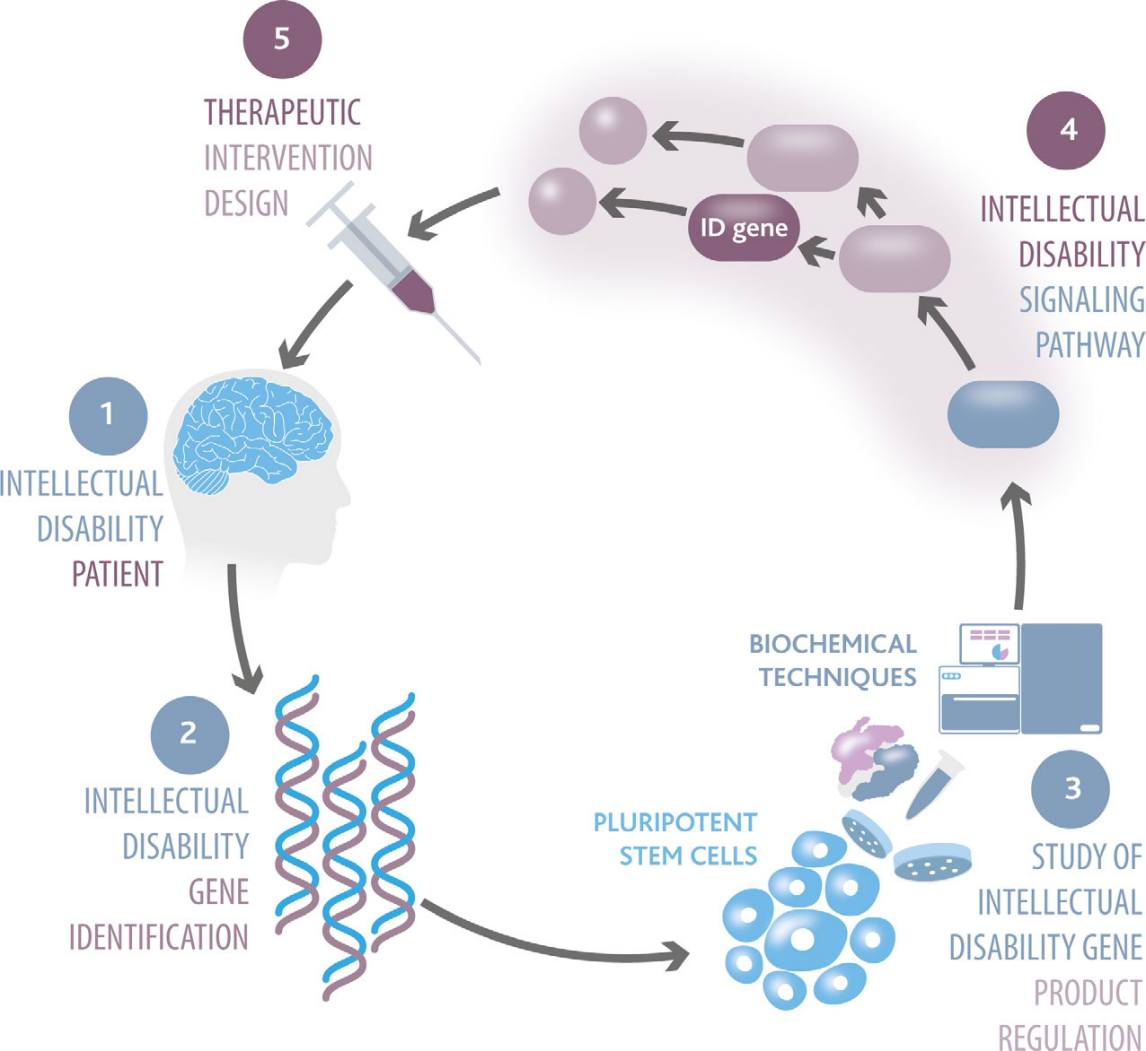
Study of signalling networks towards development of therapies for ID. ID patients are diagnosed in the clinic (1) and genetic testing is carried out to identify potential ID genes (2). We propose to combine a variety of stem cell‐based model systems with state‐of‐the‐art techniques to functionally study biochemistry and regulation of ID gene products (3). This paradigm allows dissection of novel signalling pathways that are disrupted by gene variants that cause ID (4) which will be crucial to identify molecular targets for the design of therapies focused on restoring disrupted cell signalling (5). These therapies will then serve to treat ID patients and improve cognitive function.

Emerging sequencing, genomic and annotation tools in human genetics are effective in identifying genes which cause ID driven by inherited or de novo gene variation [6, 7, 8, 9]. This is particularly true of X‐linked ID (XLID), which affects mostly males [10, 11]. XLID is estimated to account for 5–15% of all ID cases, and around 150 genes have been identified from studies of inheritance patterns in affected males and their families [10, 12]. XLID variants have been identified in genes with diverse biological functions, including those encoding signalling enzymes and transcriptional regulators [12]. Understanding the signalling networks surrounding these key components could, in principle, provide insight into how cell communication and gene expression are disrupted in patients. This in turn affords potential opportunities to therapeutically re‐establish regulatory logic and restore intellectual functioning [5, 13] (Fig. 1).

## Cell signalling and ID

Clinical genetics has identified an extensive panel of post‐translational signalling components that are mutationally disrupted in ID patients. These include protein kinases, ubiquitylation enzymes including E3 ligases and deubiquitinases (DUBs) (reviewed in [14, 15, 16]) and other regulators of post‐translational modifications such as acetylation and methylation [12]. Intriguingly, despite decades of research in this area, the products of ID genes remain largely unstudied. As a result, there is very little information on the molecular, cellular and neurodevelopmental functions of these enzymes.

Most of the functional information on XLID genes identified by clinical genetics comes from genetic disruption and phenotyping in whole organisms [17, 18, 19]. However, these models provide little or no information on the upstream signals and mechanisms that regulate these enzymes, the key substrates whose function is modified by their catalytic activities and the downstream implications for transcription, cell biology and neurological development and functioning. This illustrates a major gap between clinical genetics and molecular research that must be bridged in order to develop effective therapies. Current research approaches are often oriented towards chemical screens to identify compounds that reverse phenotypes caused by ID gene disruption. However, we propose that rationally identifying mechanisms by which ID variants disrupt signalling components and defining how these integrate with wider cell signalling networks and processes will provide opportunities to specifically modulate the function of these enzymes and signalling networks to the benefit of patients. This approach will also delineate how these gene products control normal neurodevelopment and yield valuable information on protein function and may identify hypomorphic variants that may be missed by conventional functional screens.

## Identifying specific molecular disruptions that cause ID

First, it is essential to understand the mechanisms by which ID‐associated gene variants impact on encoded signalling components. This involves initial investigation of whether the gene variant disrupts protein translation, folding or stability, which may be sufficient to classify ID variants as “likely pathogenic”. In other cases, ID‐associated gene variants may alter the biochemical properties and/or molecular function of the encoded protein in more subtle ways. These can include disrupting subcellular localisation, interaction with key functional partners such as substrates and/or co‐regulators or by directly disrupting biochemical or enzymatic activity. Investigating these functional alterations may require *a priori* knowledge of regulatory mechanisms, partner proteins and downstream substrates, which is often lacking in this field. Nevertheless, we and others have been able to exploit limited biochemical information to determine specific impacts of ID mutations on signalling proteins, thereby confirming variants as likely pathogenic.

If ID‐causing variants impact on either protein levels or specific signalling functions such as catalytic enzymatic activities, it can be inferred that unravelling the downstream substrates, gene expression programmes and cellular functions will shed light on the molecular and cellular process processes that are disrupted to cause ID. In the case of core signalling components such as protein kinases or ubiquitylation enzymes, quantitative proteomic methods for profiling changes in the phosphorylation or ubiquitylation status of proteins can reveal the key downstream substrates [20, 21]. Finally, transcriptomic profiling will identify the genetic programmes into which the emerging network feeds, enabling crucial functional insights into largely unstudied but critical neurodevelopmental signalling networks.

Research by our group and others illustrates the value of this approach. The E3 ubiquitin ligase RNF12/RLIM is mutated in patients with a syndromic form of XLID recently termed Tonne–Kalscheuer syndrome (TOKAS) [22, 23]. RNF12/RLIM TOKAS variants lead to impaired E3 ubiquitin ligase activity, which disrupts substrate ubiquitylation and proteasomal degradation [24, 25]. Previous evidence suggests that RNF12 ubiquitylates transcriptional regulators, most prominently the developmental transcriptional controller REX1/ZFP42 [26, 27, 28]. Our investigations find that REX1 may be a RNF12 substrate that is highly relevant for development of TOKAS [29]. In the presence of RNF12 TOKAS variants, REX1 ubiquitylation and degradation are impaired, leading to REX1 accumulation in cells [24]. This in turn leads to deregulation of an RNF12‐dependent neurodevelopmental gene expression programme [29] and aberrant neural differentiation [24]. In principle, disruption of this signalling pathway for neurodevelopmental regulation by RNF12/RLIM TOKAS variants could drive ID in TOKAS patients, providing detailed molecular insight into the mechanisms underpinning this ID syndrome.

This approach is further illustrated in elegant work by Werner and colleagues, who showed that the deubiquitylating enzyme OTUD5 is mutated in a form of ID known as LINKED syndrome [30]. This study identified a series of OTUD5 variants in ID patients, although the substrates of OTUD5 were not known. Quantitative proteomics pinpointed a cohort of key chromatin modifiers, including ARID1A/B, HDAC2, HCF1 and UBR5 that are direct substrates of OTUD5 which work in combination to regulate expression of neurodevelopmental genes [30]. This again provides an elegant molecular framework to explain the development of ID in LINKED syndrome patients with OTUD5 gene variants.

Importantly, these examples also illustrate how new molecular insights into signalling defects in ID can reveal potential therapeutic strategies. In the case of RNF12/RLIM signalling that is dysregulated in TOKAS, restoration of REX1 substrate degradation is predicted to normalise the expression of neurodevelopmental genes in TOKAS patients. In the case of OTUD5, restoring deubiquitylation activity, perhaps by destruction of the cognate E3 ubiquitin ligase(s) which ubiquitylate OTUD5 substrate proteins involved in chromatin organisation, could be employed to re‐establish neurodevelopmental gene expression. In principle, each of these objectives could be achieved via targeted protein degradation approaches. These include proteolysis targeting chimeras (PROTACs) [31], which are heterobifunctional molecules that chemically juxtapose a target protein and a non‐cognate E3 ubiquitin ligase, leading to neomorphic target ubiquitylation and destruction. Therefore, understanding the key molecular processes that are disrupted in pathways downstream of ID gene variants can enable personalised and/or targeted approaches to restore signalling and potentially normalise neurodevelopmental gene expression.

## Comprehensively mapping signalling networks that are disrupted in ID

Initial focus is to address the key downstream molecular processes that are disrupted by ID gene variants. However, upstream regulation of signalling components that are mutated in ID will be highly relevant to gain a complete understanding of the molecular and cellular processes that are disrupted to cause ID. Furthermore, genes encoding these key upstream regulatory components may themselves be mutated in ID patients with overlapping clinical features to introduce further ID‐associated gene variants. We suggest that iteratively mapping ID signalling networks in this way will eventually provide a complete picture of the regulatory logic that is disrupted in various forms of ID and will in turn reveal further potential ID genes and signalling nodes that may be exploited in therapeutics (Fig. 1).

Our own research again supports this notion. In searching for upstream regulators of the E3 ubiquitin ligase RNF12/RLIM, which is mutated in TOKAS ID, we found using chemical inhibitor and recombinant kinase screens that the splicing factor kinase SRSF protein kinase (SRPK) directly phosphorylates RNF12, leading to activation and nuclear anchoring [29]. As a result, SRPK phosphorylation of RNF12 plays a key role in regulation of RNF12 substrate ubiquitylation and in regulation of neurodevelopmental genes [29]. Therefore, SRPK is directly linked to downstream processes that are relevant for ID. This prompted a search of databases reporting identification of gene variants associated with ID phenotypes to determine whether the SRPK gene family is also disrupted in these disorders. Indeed, *SRPK2*, which is highly expressed in the human brain, is deleted in ID patients, while the X‐linked *SRPK3,* which is expressed in a very specific subset of human neurons, is frequently amplified, deleted and mutated in patients. Investigation of the biochemical impact of *SRPK3* ID‐associated point mutations suggests that a subset display severely impaired kinase activity [29]. Taken together, these findings suggest that SRPK lies upstream of RNF12 in an emerging ID signalling pathway, of which multiple components are mutated in patients with overlapping clinical features. Based on this paradigm, future studies will involve high‐throughput ‐omics approaches to map networks of post‐translational modifications, comprehensively identifying key nodes and shedding light on the signalling networks that are disrupted in ID patients.

## Understanding ID signalling in context

One of the major challenges in the ID field is contextual, that is, to effectively combine study of the relevant tissues in the nervous system with a tractable experimental system to unravel signalling networks in molecular detail. Standard laboratory cell culture systems are tractable but are far removed from the developing nervous system. Therefore, developmental components may not be expressed and/or regulation and function are not conserved. Animal models can provide an important description of how relevant signalling systems are expressed and wired in the developing nervous system. However, whole organisms are not amenable to in‐depth analysis and acute signalling perturbation, and neurological phenotypes are frequently marginal and/or difficult to detect.

In our experience, this gap is most effectively bridged using pluripotent stem cell models including mouse embryonic stem cells, but particularly human embryonic stem cells or patient‐derived human induced pluripotent stem cells [32]. This enables researchers to perform detailed molecular and biochemical analysis in a tractable mammalian/human neuronal cell system while simultaneously garnering insights into developmental phenotypes that may be relevant for ID [33, 34]. Furthermore, the capacity of pluripotent stem cells to differentiate into any cell type in the body including adult neural lineages provides a powerful tool to directly investigate how signalling networks engage the neurodevelopmental gene expression programmes that are exquisitely controlled to ensure correct neurological development and functioning [33]. Moreover, the advent of organoid‐based technologies affords us future opportunities to investigate how signalling pathways control development of increasingly sophisticated and complete human “mini‐brains” grown in the laboratory [35]. When combined with single‐cell RNA‐sequencing, organoids could provide unprecedented insight into the impact of ID gene variants on temporal and region‐specific neurodevelopmental trajectories [36]. In short, pluripotent stem cells provide a surprisingly direct route by which to determine how complex molecular connections at the biochemical level impact on neurodevelopmental gene expression and therefore development of the human nervous system.

## Therapeutic opportunities

What are the immediate therapeutic opportunities afforded by dissecting the signalling networks underpinning ID? A major benefit of research in this area is that signalling enzymes (and protein kinases in particular) have been exploited as therapeutic targets for decades, with scores of small molecule kinase inhibitors approved for clinical use. Furthermore, phosphatase inhibitors show growing promise as therapeutic targets that may reverse defects in kinase signalling caused by ID variants that disrupt kinase activity. In the ubiquitin field, PROTACs that are entering clinical trials induce or restore degradation of key therapeutic targets [37], and as discussed earlier could be exploited to normalise levels of protein substrates that accumulate in the presence of variants that disrupt activity of the cognate E3 ubiquitin ligase. Finally, small molecule DUB inhibitors targeting distinct mechanistic aspects of enzymatic function [38] have shown recent clinical promise [39] and may enable modulation of ubiquitin‐dependent signalling networks to modulate protein degradation in a similar manner. Excellent progress in the area of therapeutic tools and development pipelines to target the relevant signalling networks in ID suggests these will form important resources to ultimately benefit patients.

## Future challenges

An ever‐present challenge in this field is the ability to identify causal gene variants that strongly segregate with ID patients. In our view, this requires rigorous genetic analysis in combination with biochemical investigation to functionally characterise patient variants and assign causality. Although many ID genes are identified as inherited or *de novo* germline variants, somatic mutations during brain development must be considered as these can contribute to neurodevelopmental disorders [40]. ID gene identification will provide a robust starting point for mapping pathways and networks which are crucial for intellectual function. Although the primary objective will be dissection of discrete signalling pathways, understanding the links between distinct components and pathways that are mutated in related syndromes could require new systems biology and bioinformatic tools for network integration and annotation together with simulations/predictions of protein functional association and how these are disrupted by ID gene variants. Once the information flow within a signalling network is elucidated, it will then be possible to define nodes within that network that are appropriate for therapeutic intervention.

A key question that remains is whether the neurological manifestations of ID disorders are generally reversible in children and adult patients, or whether treatments or gene editing must be performed on embryos prior to embryonic neurological development. The latter may be true in many cases. However, in support of the former is the first example of mechanism‐based therapy for ID caused by phenylketonuria (PKU) [41], where careful restriction of tyrosine uptake leads to marked improvement in intellectual functioning [42]. Recent investigations also address the reversibility of other genetic neurodevelopmental disorders by defining the relevance of ID genes for neurological function and determining whether phenotypes are reversed by restoring ID gene function. Thus far, adult neurological and behavioural phenotypes have been shown to be at least partially reversible in mouse models of Rett syndrome caused by deletions in MeCP2 [43], SYNGAP1 haploinsufficiency [44], SHANK3‐induced autism [45] and CDKL5 deficiency disorder [46]. Furthermore, clinical studies on Fragile‐X syndrome, which is caused by inactivation of the mRNA binding protein and translational regulator FMR1, show promise in reversing the effects of FMR1 disruption [47, 48]. This suggests a potential postnatal therapeutic window for reversal of neurological dysfunction associated with at least a sub‐set of specific ID disorders. It is important to note that these interventions were developed based on a profound understanding of the molecular mechanisms underlying the disorder. If the same is true of other IDs caused by disrupted cell signalling, then signalling network mapping followed by target validation in the developing nervous system at the developmental stage of interest may enable mechanism‐based targeted chemical interventions to normalise signalling and reverse neurological phenotypes.

This raises a final challenge of how to effectively model ID syndromes to provide pre‐clinical validation of therapeutic strategies. In our view, it is essential to understand both molecular signalling disruptions and high‐level neurological pathophysiology in order to develop effective therapies. Current experimental systems in the ID field each have major drawbacks. Human‐derived pluripotent stem cells and neuronal differentiation systems suffer the obvious limitation of being an overly simplified *in vitro* system that is inappropriate for pre‐clinical testing. In the longer term, this may be resolved using sophisticated stem cell‐derived brain organoids, which may be sufficiently complex to reveal key physiological features of ID disorders. As discussed previously, some animal models of ID gene variants have shown utility for measuring ID phenotypes and therefore could provide pre‐clinical validation. However, robust ID related phenotypes are not always obvious or even present making it very difficult to identify and measure impact of potential therapeutics on developmental, structural or behavioural defects of the nervous system. Therefore, the challenge remains to identify model systems that can provide tractable and quantifiable ID phenotypes, as this remains essential to enable pre‐clinical testing of therapeutic interventions.

## Conflict of interest

The authors declare no conflict of interest.

## Author contributions

GMF and FB conceived, drafted, edited and revised the manuscript.

## Data Availability

Data sharing is not applicable to this article as no new data were created in this study.

## References

[1] Panca M , Buszewicz M , Strydom A , Hassiotis A , Welch CA , Hunter RM . Resource use and cost of annual health checks in primary care for people with intellectual disabilities. J Intellect Disabil Res. 2019;63:233–43. 30461105 10.1111/jir.12569 PMC6451619

[2] Schalock RL , Luckasson RA , Shogren KA , Borthwick‐Duffy S , Bradley V , Buntinx WHE , et al. The renaming of mental retardation: understanding the change to the term intellectual disability. Intellect Dev Disabil. 2007;45:116–24. 17428134 10.1352/1934-9556(2007)45[116:TROMRU]2.0.CO;2

[3] Leonard H , Wen X . The epidemiology of mental retardation: challenges and opportunities in the new millennium. Ment Retard Dev Disabil Res Rev. 2002;8:117–34. 12216056 10.1002/mrdd.10031

[4] McKenzie K , Milton M , Smith G , Ouellette‐Kuntz H . Systematic review of the prevalence and incidence of intellectual disabilities: current trends and issues. Curr Dev Disord Rep. 2016;3:104–15.

[5] Picker JD , Walsh CA . New innovations: therapeutic opportunities for intellectual disabilities. Ann Neurol. 2013;74:382–90. 24038210 10.1002/ana.24002 PMC3876407

[6] Topper S , Ober C , Das S . Exome sequencing and the genetics of intellectual disability. Clin Genet. 2011;80:117–26. 21627642 10.1111/j.1399-0004.2011.01720.x PMC4343531

[7] Ellison JW , Rosenfeld JA , Shaffer LG . Genetic basis of intellectual disability. Annu Rev Med. 2013;64:441–50. 23020879 10.1146/annurev-med-042711-140053

[8] Chiurazzi P , Pirozzi F . Advances in understanding – genetic basis of intellectual disability. F1000Res. 2016;5:599. 10.12688/f1000research.7134.1 PMC4830215 27127621

[9] de Ligt J , Willemsen MH , van Bon BWM , Kleefstra T , Yntema HG , Kroes T , et al. Diagnostic exome sequencing in persons with severe intellectual disability. N Engl J Med. 2012;367:1921–9. 23033978 10.1056/NEJMoa1206524

[10] Ropers H‐H , Hamel BCJ . X‐linked mental retardation. Nat Rev Genet. 2005;6:46–57. 15630421 10.1038/nrg1501

[11] Zechner U , Wilda M , Kehrer‐sawatzki H , Vogel W , Hameister H , Hameister H . A high density of X‐linked genes for general cognitive ability: a run‐away process shaping human evolution? Trends Genet. 2001;17:697–701. 11718922 10.1016/s0168-9525(01)02446-5

[12] Neri G , Schwartz CE , Lubs HA , Stevenson RE . X‐linked intellectual disability update 2017. Am J Med Genet Part A. 2018;176:1375–88. 29696803 10.1002/ajmg.a.38710 PMC6049830

[13] O’Connor TP , Crystal RG . Genetic medicines: treatment strategies for hereditary disorders. Nat Rev Genet. 2006;7:261–76. 16543931 10.1038/nrg1829

[14] George AJ , Hoffiz YC , Charles AJ , Zhu Y , Mabb AM . A comprehensive atlas of E3 ubiquitin ligase mutations in neurological disorders. Front Genet. 2018;9:1–17. 29491882 10.3389/fgene.2018.00029 PMC5817383

[15] Basar MA , Beck DB , Werner A . Deubiquitylases in developmental ubiquitin signaling and congenital diseases. Cell Death Differ. 2021;28:538–56. 33335288 10.1038/s41418-020-00697-5 PMC7862630

[16] Folci A , Mirabella F , Fossati M . Ubiquitin and ubiquitin‐like proteins in the critical equilibrium between synapse physiology and intellectual disability. Eneuro. 2020;7:ENEURO.0137‐20.2020. 10.1523/ENEURO.0137-20.2020 PMC7544190 32719102

[17] Leslie EJ . Embracing human genetics: a primer for developmental biologists. Development. 2020;147:dev191114. 32616565 10.1242/dev.191114 PMC7338264

[18] Scorza CA , Cavalheiro EA . Animal models of intellectual disability: towards a translational approach. Clinics. 2011;66:55–63. 21779723 10.1590/S1807-59322011001300007 PMC3118438

[19] Homberg JR , Kyzar EJ , Nguyen M , Norton WH , Pittman J , Poudel MK , et al. Understanding autism and other neurodevelopmental disorders through experimental translational neurobehavioral models. Neurosci Biobehav Rev. 2016;65:292–312. 27048961 10.1016/j.neubiorev.2016.03.013

[20] Kirkpatrick DS , Denison C , Gygi SP . Weighing in on ubiquitin: the expanding role of mass‐spectrometry‐based proteomics. Nat Cell Biol. 2005;7:750–7. 16056266 10.1038/ncb0805-750 PMC1224607

[21] Rogne M , Taskén K . Cell signalling analyses in the functional genomics era. N Biotechnol. 2013;30:333–8. 23369868 10.1016/j.nbt.2013.01.003

[22] Tønne E , Holdhus R , Stansberg C , Stray‐Pedersen A , Petersen K , Brunner HG , et al. Syndromic X‐linked intellectual disability segregating with a missense variant in RLIM. Eur J Hum Genet. 2015;23:1652–6. 25735484 10.1038/ejhg.2015.30 PMC4795204

[23] Hu H , Haas SA , Chelly J , Van Esch H , Raynaud M , de Brouwer APMM , et al. X‐exome sequencing of 405 unresolved families identifies seven novel intellectual disability genes. Mol Psychiatry. 2016;21:133–48. 25644381 10.1038/mp.2014.193 PMC5414091

[24] Bustos F , Segarra‐Fas A , Chaugule VK , Brandenburg L , Branigan E , Toth R , et al. RNF12 X‐linked intellectual disability mutations disrupt E3 ligase activity and neural differentiation. Cell Rep. 2018;23:1599–611. 29742418 10.1016/j.celrep.2018.04.022 PMC5976579

[25] Frints SGM , Ozanturk A , Rodríguez Criado G , Grasshoff U , de Hoon B , Field M , et al. Pathogenic variants in E3 ubiquitin ligase RLIM/RNF12 lead to a syndromic X‐linked intellectual disability and behavior disorder. Mol Psychiatry. 2019;24:1748–68. 29728705 10.1038/s41380-018-0065-x

[26] Gontan C , Achame EM , Demmers J , Barakat TS , Rentmeester E , van IJcken W , et al. RNF12 initiates X‐chromosome inactivation by targeting REX1 for degradation. Nature. 2012;485:386–90. 22596162 10.1038/nature11070

[27] Gontan C , Mira‐Bontenbal H , Magaraki A , Dupont C , Barakat TS , Rentmeester E , et al. REX1 is the critical target of RNF12 in imprinted X chromosome inactivation in mice. Nat Commun. 2018;9:4752. 30420655 10.1038/s41467-018-07060-w PMC6232137

[28] Zhang L , Huang H , Zhou F , Schimmel J , Pardo CGG , Zhang T , et al. RNF12 controls embryonic stem cell fate and morphogenesis in zebrafish embryos by targeting Smad7 for degradation. Mol Cell. 2012;46:650–61. 22560923 10.1016/j.molcel.2012.04.003

[29] Bustos F , Segarra‐Fas A , Nardocci G , Cassidy A , Antico O , Davidson L , et al. Functional diversification of SRSF protein kinase to control ubiquitin‐dependent neurodevelopmental signaling. Dev Cell. 2020;55:629–47.e7. 33080171 10.1016/j.devcel.2020.09.025 PMC7725506

[30] Beck DB , Basar MA , Asmar AJ , Thompson JJ , Oda H , Uehara DT , et al. Linkage‐specific deubiquitylation by OTUD5 defines an embryonic pathway intolerant to genomic variation. Sci Adv. 2021;7:eabe2116. 33523931 10.1126/sciadv.abe2116 PMC7817106

[31] Burslem GM , Crews CM . Small‐molecule modulation of protein homeostasis. Chem Rev. 2017;117:11269–301. 28777566 10.1021/acs.chemrev.7b00077

[32] Takahashi K , Okita K , Nakagawa M , Yamanaka S . Induction of pluripotent stem cells from fibroblast cultures. Nat Protoc. 2007;2:3081–9. 18079707 10.1038/nprot.2007.418

[33] Telias M , Ben‐Yosef D . Modeling neurodevelopmental disorders using human pluripotent stem cells. Stem Cell Rev Rep. 2014;10:494–511. 24728983 10.1007/s12015-014-9507-2

[34] Freel BA , Sheets JN , Francis KR . iPSC modeling of rare pediatric disorders. J Neurosci Methods. 2020;332:108533. 31811832 10.1016/j.jneumeth.2019.108533 PMC7310918

[35] Baldassari S , Musante I , Iacomino M , Zara F , Salpietro V , Scudieri P . Brain organoids as model systems for genetic neurodevelopmental disorders. Front Cell Dev Biol. 2020;8:590119. 33154971 10.3389/fcell.2020.590119 PMC7586734

[36] Kanton S , Boyle MJ , He Z , Santel M , Weigert A , Sanchís‐Calleja F , et al. Organoid single‐cell genomic atlas uncovers human‐specific features of brain development. Nature. 2019;574:418–22. 31619793 10.1038/s41586-019-1654-9

[37] Qi SM , Dong J , Xu ZY , Cheng XD , Zhang WD , Qin JJ . PROTAC: an effective targeted protein degradation strategy for cancer therapy. Front Pharmacol. 2021;12:692574. 34025443 10.3389/fphar.2021.692574 PMC8138175

[38] Lange SM , Armstrong LA , Kulathu Y . Deubiquitinases: from mechanisms to their inhibition by small molecules. Mol Cell. 2022;82:15–29. 34813758 10.1016/j.molcel.2021.10.027

[39] Antao AM , Tyagi A , Kim KS , Ramakrishna S . Advances in deubiquitinating enzyme inhibition and applications in cancer therapeutics. Cancers (Basel). 2020;12:1579. 32549302 10.3390/cancers12061579 PMC7352412

[40] McRae JF , Clayton S , Fitzgerald TW , Kaplanis J , Prigmore E , Rajan D , et al. Prevalence and architecture of de novo mutations in developmental disorders. Nature. 2017;542:433–8. 28135719 10.1038/nature21062 PMC6016744

[41] Fölling A . Über ausscheidung von phenylbrenztraubensäure in den harn als stoffwechselanomalie in verbindung mit imbezillität. Hoppe Seylers Z Physiol Chem. 1934;227:169–81.

[42] Kaufman S . Phenylketonuria: biochemical mechanisms. Adv Neurochem. 1977;2:1–132.

[43] Guy J , Gan J , Selfridge J , Cobb S , Bird A . Reversal of neurological defects in a mouse model of Rett syndrome. Science (80‐). 2007;315:1143–7. 10.1126/science.1138389 PMC7610836 17289941

[44] Creson TK , Rojas C , Hwaun E , Vaissiere T , Kilinc M , Jimenez‐Gomez A , et al. Re‐expression of SynGAP protein in adulthood improves translatable measures of brain function and behavior. Elife. 2019;8:1–19. 10.7554/eLife.46752 PMC6504227 31025938

[45] Mei Y , Monteiro P , Zhou Y , Kim J‐A , Gao X , Fu Z , et al. Adult restoration of Shank3 expression rescues selective autistic‐like phenotypes. Nature. 2016;530:481–4. 26886798 10.1038/nature16971 PMC4898763

[46] Terzic B , Davatolhagh MF , Ho Y , Tang S , Liu Y‐T , Xia Z , et al. Temporal manipulation of Cdkl5 reveals essential postdevelopmental functions and reversible CDKL5 deficiency disorder‐related deficits. J Clin Invest. 2021;131:e143655. 34651584 10.1172/JCI143655 PMC8516470

[47] Berry‐Kravis E . Mechanism‐based treatments in neurodevelopmental disorders: fragile X syndrome. Pediatr Neurol. 2014;50:297–302. 24518745 10.1016/j.pediatrneurol.2013.12.001

[48] Berry‐Kravis EM , Lindemann L , Jønch AE , Apostol G , Bear MF , Carpenter RL , et al. Drug development for neurodevelopmental disorders: lessons learned from fragile X syndrome. Nat Rev Drug Discov. 2018;17:280–99. 29217836 10.1038/nrd.2017.221 PMC6904225

